# Communicating Healthcare Economic and Pre-approval Information With Healthcare Decision-Makers: Opportunities Following the 21st Century Cures Act and FDA Guidance

**DOI:** 10.3389/fpubh.2018.00304

**Published:** 2018-11-14

**Authors:** Alexander Niyazov, Denise Lenci

**Affiliations:** Mosaic Group, New York, NY, United States

**Keywords:** healthcare economic information, pre-approval, pharmaceutical exchange, FDAMA 114, 21st Century Cures Act

## Abstract

With rising US healthcare costs, population health decision-makers have expressed interest in receiving pre-approval information to help with the budgeting and forecasting needed to accommodate pharmaceutical and medical device launches. Additionally, there has been a strong emphasis placed on the economics and quality of new products. Manufacturers have historically been reluctant to share pre-approval or healthcare economic information (HCEI) due to unclear regulatory guidance for these types of communications. The 21st Century Cures Act, as well as the June 2018 FDA guidance on payor communications have more clearly defined guardrails to communicate this information. This paper provides insights on how to optimize this new guidance and facilitate robust and compliant conversations with decision-makers.

## Background

As US healthcare costs continue to rise, decision-makers involved in population health management have put a greater focus on the economics and quality of pharmaceuticals and medical devices. In response to this renewed value focus, professional societies have put forward value frameworks. In March of 2014, the American College of Cardiology and the American Heart Association issued guidance on value considerations including Cost/QALY thresholds (1). Around the same time, the Institute for Clinical and Economic Review (ICER), which conducts formal Cost/QALY economic analyses, emerged as an influential force in the discussion of value assessments (2). While pharmaceutical manufacturers have made strides in responding to value assessment considerations, the industry has historically been reluctant to share healthcare economic information (HCEI) with formulary decision-makers due to unclear regulatory guidance for these types of communications.

Given that the US is becoming more value-focused, it may come as no surprise that payors have expressed interest in receiving information on pipeline agents. Many payors were blindsided by the launch of the hepatitis C drugs and the impact this had on their budgets (3). They claim that waiting for FDA approval leaves them too little time to initiate the planning, forecasting, and budgeting that is needed to accommodate a new product launch. Payors have suggested that the ideal timeframe to have these conversations is 12–18 months prior to approval, which would allow for appropriate planning and forecasting decisions (4).

## 21st century cures act and FDA guidance—healthcare economic information

Prior to 1997, manufacturers had to demonstrate “substantial evidence,” usually from two randomized controlled trials, in order to promote HCEI (5). In 1997, Section 114 of the Food and Drug Modernization Act (FDAMA) was enacted, easing the evidentiary standard from “substantial evidence” to “competent and reliable scientific evidence” (CARSE) (6). The goal of FDAMA 114 was to facilitate the exchange of HCEI between pharmaceutical manufacturers and formulary committees or similar entities. However, the regulatory language did not establish clear guardrails for this information exchange.

In July of 2016, the Pharmaceutical Research and Manufacturers of America (PhRMA) and the Biotechnology Innovation Organization (BIO) released their “Principles on Responsible Sharing of Truthful and Non-Misleading Information about Medicines with Health Care Professionals and Payers” (7). The goal of these principles was to guide the industry in promotional communications with these two distinct audiences. Around the same time, the Academy of Managed Care Pharmacy (AMCP) convened a panel of diverse stakeholders to develop and share with the FDA considerations for updating FDAMA 114 based on the current decision-making landscape (8).

In the absence of clear regulatory guidance, pharmaceutical manufacturers created their own policies for sharing HCEI. Some provide HCEI in response to unsolicited medical requests only; some restrict dissemination of this information to medical affairs staff (such as medical science liaisons) or to health economics and outcomes research (HEOR) staff; while others allow payor account managers to present limited HCEI. In some cases, there is a strict “firewall” between managed care account teams and HEOR and medical affairs. This variation creates discontinuity in payor communications across the pharmaceutical industry, but also within individual manufacturers.

In December of 2016, Congress passed the 21st Century Cures Act, which clarified some of the language surrounding HCEI under FDAMA 114. In particular, Section 3037 (9):

1. Clarified, to some degree, the definition of HCEI and the types of economic analyses that may be included for promotional purposes.
  - “Any analysis (including the clinical data, inputs, clinical or other assumptions, methods, results, and other components underlying or comprising the analyses) that identifies, measures, or describes the economic consequences, which may be based on the separate or aggregated clinical consequences of the represented health outcomes, of the use of a drug. Such analysis may be comparative to the use of another drug, to another health care intervention or to no intervention.”
2. Changed “Directly Related” to an approved indication to “Relates” to an approved indication.
  - Under FDAMA 114 there was ambiguity surrounding what is considered directly related to an approved indication. This led some manufacturers to take a very conservative approach on projecting economic benefits based on real-world data for fear that the patient populations in real-world studies may be different from those studied in clinical trials. Removal of the word “directly” gives manufacturers more opportunity to include real-world studies, as well as the outcomes assessed. For example, one could make the argument that for a hepatitis C drug, an economic evaluation may look at the cost savings from a liver transplant, which is a complication “related” to hepatitis C and that may occur in patients not receiving treatment or receiving suboptimal treatment.
3. Required a disclaimer surrounding differences between the economic evaluation and the product labeling.
  - That such a statement is required alerts us that there are different evidentiary requirements for regulatory approval vs. formulary evaluation. Considering that clinical trials have strict inclusion/exclusion criteria, the findings in clinical trials may not be representative of the real world. Furthermore, as products become more mature, formulary decision-makers may want to substantiate efficacy from real world-data.
4. Provided clarification on the audience that may receive HCEI.
  - This audience includes formulary committees and other similar entities.

In January 2017, the FDA issued draft guidance on Drug and Device Manufacturer Communications with Payors, Formulary Committees and Similar Entities, and in June 2018, the Agency issued its final guidance (10, 11). The final guidance further expands the audience that can receive HCEI to include drug information centers, technology assessment panels, pharmacy benefit managers, and other multidisciplinary entities, and it clarifies that individuals who serve multiple roles involving direct patient care and population health management, may receive HCEI when they are carrying out their responsibilities involving formulary management. The FDA guidance also provides examples of the types of healthcare economic analyses that relate to an approved indication.

## Pre-approval communication and FDA guidance

Pharmaceutical manufacturers also have encountered limitations in communicating about investigational agents. This language in Title 21 Code of Federal Regulations Section 312.7 limits the promotion of investigational agents (12):

“*Promotion of an investigational new drug*. A sponsor or investigator, or any person acting on behalf of a sponsor or investigator, shall not represent in a promotional context that an investigational new drug is safe or effective for the purposes for which it is under investigation or otherwise promote the drug. This provision is not intended to restrict the full exchange of scientific information concerning the drug, including dissemination of scientific findings in scientific or lay media. Rather, its intent is to restrict promotional claims of safety or effectiveness of the drug for a use for which it is under investigation and to preclude commercialization of the drug before it is approved for commercial distribution.”

In 2012, the Food and Drug Administration Safety and Innovation Act (FDASIA) was passed and allowed accelerated approval for medicines that “treat a serious or life-threatening condition” if “preliminary clinical evidence indicates that the drug may demonstrate substantial improvement over existing therapies” (13). Because FDA approval could occur prior to publication of clinical trial data, payors could find themselves having to make formulary decisions without complete information. The AMCP became concerned about this challenge and, in March 2016, convened a panel of diverse stakeholders to develop recommendations on pre-approval communication for the FDA (8). Subsequently, the FDA's January 2017 draft guidance on Drug and Device Manufacturer Communications with Payors, Formulary Committees and Similar Entities included a section focused on pre-approval communications (10). Notably, the draft guidance did not address communications about unapproved uses of approved/cleared products. This was later addressed when the FDA released its final guidance document in June 2018 (11).

## Implementing the new guidance for HCEI

With the passage of the 21st Century Cures Act, as well as issuance of FDA guidance, it is clear there is more opportunity for a dialogue between manufacturers and payors about HCEI information. The FDA has recognized that payors are sophisticated individuals who need information to guide their decision-making process. Additionally, the agency has stated that communicating this information is a low public health risk if done in accordance with the agency's guidance. It appears that the FDA acknowledges that the evidentiary standards for formulary decision-making are different than for regulatory approval.

In response to the new guidance many pharmaceutical companies are re-evaluating their own policies and procedures. Some have begun to incorporate more extensive HCEI into their payor promotional materials and to share pre-approval information. The following are suggestions for optimizing application of the FDA guidance within a pharmaceutical or medical device company:

1. **Engage medical, regulatory, legal, HEOR, and marketing teams to review the guidance and develop internal rules for what HCEI can be included in promotional materials**. In addition to listing examples of HCEI analyses that relate to an approved indication, the FDA guidance directs that “firms should include appropriate background and contextual information when disseminating HCEI,” and goes on to discuss what would comprise a “balanced and complete presentation.” Manufacturers may benefit from careful review of these recommendations and creation of a specific policy for their own medical affairs, HEOR, and managed care marketing departments.
2. **Create open dialogue between managed care marketing and HEOR teams**. Not all HEOR studies are candidates for promotional use, therefore discussion between market access and HEOR is critical in order to manage expectations about data dissemination. It may be good practice to have these interactions prior to the study initiation to ensure the study is relevant to the market landscape and can be used in payor communications. If there is any concern about the design of a proposed study, an independent HEOR expert reviewer may help ensure that the standard of “CARSE” is met.
3. **Determine who will be empowered to communicate HCEI and in what format**. The FDA guidance acknowledges that HCEI can be presented in a variety of ways, including “an evidence dossier, a reprint of a publication from a peer-reviewed journal, a software package comprising a model with a user manual, a budget-impact model, a slide presentation, or a payor brochure.” These are very different types of tools that require different knowledge and skill sets to communicate effectively. While it may vary among organizations, healthcare economic exchanges traditionally have been handled by medical affairs staff. Given that the new guidance allows for promotional use of these materials, manufacturers may want account managers to share HCEI, as well. Regardless of who is delivering the presentation, it is important that organizations establish processes and provide the necessary training to their field teams in order to create a meaningful dialogue with payors.

While the above recommendations apply to both drug and devices manufacturers, it should be noted that there is a distinct difference in the approval of such pieces. Communication pieces that feature HCEI for approved drugs are considered promotional materials and must be submitted to the FDA using Form FDA 2253. In contrast, device manufacturers do not need to submit HCEI-containing pieces to the FDA for review. This is an important distinction as it impacts timelines for when these materials will be available for use.

## Implementing the new guidance for pre-approval communications

Similar to HCEI, manufacturers are trying to identify the best strategies to apply this new guidance in the pre-approval time period. Engaging in pre-approval communications provides opportunities for manufacturers to cultivate relationships with their customers, raise awareness about upcoming product launches, and potentially ease formulary reviews and protocol placements. For payors, such communications enable them to forecast resources in anticipation of new launches.

The above, of course, is contingent upon how well manufacturers communicate the information payors are seeking. For example, the FDA guidance recommends that manufacturers include product pricing information when discussing pre-approval information. This may be a sensitive topic for manufacturers to discuss as the price may be contingent upon final efficacy data, as well as the competitive landscape. Manufacturers may be more comfortable offering payors general guidance on pricing such as a price range or disclosing whether they are forecasting to be at price parity or charge a premium to the current standard of care.

**Figure 1:**
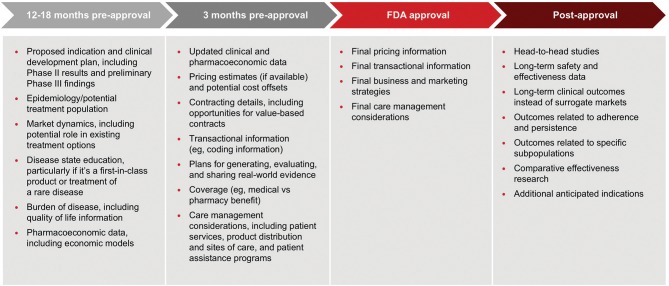
An AMCP payer/pharma workshop developed recommendations on what information payers want to receive from manufacturers and when it should be delivered. These recommendations were reported at the AMCP annual meeting in April 2018.

It remains to be seen to what extent manufacturers will feel comfortable disclosing pricing information, however manufacturers should consider the type of information payors are seeking and when they want to receive it when planning pre-approval communications. In early 2018, AMCP convened a task force of pharmaceutical manufacturers and payors to consider this topic. Figure 1 below summarizes the task force recommendations as described at the AMCP annual meeting in April 2018 (14).

This framework offers a good starting point for pharmaceutical manufacturers to develop their own policies. In fact, many companies are beginning to implement internal guidelines and undertake staff training. As with HCEI, key considerations include:

□ **Who may conduct pre-approval communications with payers?** The FDA guidance does not address this question. However, the AMCP taskforce did consider it and suggested that the job title or function is not the critical factor; their recommendation is that staff who deliver the information should be able to knowledgably and effectively communicate clinical, economic, and outcomes data related to the product (14). At least one pharmaceutical company has developed a policy that delineates information that can be communicated by account managers vs. medical staff. They are reserving discussions about clinical trial results and economic models for MSLs and HEOR representatives.
□ **Who is the audience and how to ensure that *only* appropriate customers receive these communications?** The FDA guidance identifies the audience who may receive pre-approval information as the same as the audience for HCEI. Yet, for some companies that develop hospital/health system products there is caution about the distinction between an HCP's role as a prescriber vs. “carrying out their professional responsibilities for selection of drugs for coverage or reimbursement for a payor, formulary committee, or similar entity.” In addition, there may be challenges in limiting communications to “payors.” Manufacturers are developing processes to accurately identify and document with whom they are communicating pre-approval information.
□ **What information can be shared and when?** This FDA guidance is quite clear about what information can be shared, but is silent regarding timing. [The AMCP taskforce suggests that manufacturers should engage with payors at least 24 months in advance of FDA approval to seek their input about clinical trial design (14)]. It appears that many manufactures are engaging payors in dialogue between 12 and 6 months prior to anticipated approval, with creation of communication plans, and materials beginning some months before then.
□ **What form can these communications take?** Again, the FDA guidance does not address this question directly. Manufacturers will need to determine how they want to communicate pre-approval, but currently the main method seems to be in-person presentations with no print materials left behind. This is due, in part, to concerns about the information ending up with non-payor audiences and the possibility that data will change during the drug development process. AMCP is working to create compliant pathways to share pre-approval information with its members, as are other organizations, which may allow for a wider audience for these important communications.

## Looking ahead

As it relates to HCEI, it is important to distinguish that the FDA guidance builds upon the 21st Century Cures Act, which was passed by Congress. In contrast, Congress did not pass any recent legislation including pre-approval communication. AMCP is advocating with Congress for passage of the Pharmaceutical Information Exchange (PIE) Act, which would further support manufacturers' sharing pre-approval clinical and economic information with healthcare decision- makers. As of January 2018, the PIE Act was passed by the US House Committee on Energy and Commerce's Subcommittee on Health. The bill is now before the full Committee for consideration (15).

## Author contributions

AN and DL contributed to the writing and editing of the manuscript.

### Conflict of interest statement

At the time this manuscript was written, AN and DL were employed by Mosaic Group a market access communications agency that is part of the FCBHealth network. AN is no longer employed at Mosaic Group and is now employed at Pfizer.
